# Population receptive field (pRF) measurements of chromatic responses in human visual cortex using fMRI

**DOI:** 10.1016/j.neuroimage.2017.11.022

**Published:** 2018-02-15

**Authors:** Lauren E. Welbourne, Antony B. Morland, Alex R. Wade

**Affiliations:** a Department of Psychology, University of York, Heslington, YO10 5DD, UK; b York NeuroImaging Centre, The Biocentre, York Science Park, Heslington, YO10 5NY, UK

**Keywords:** pRF, Chromatic pathways, fMRI, Spatial frequency, Luminance, Color

## Abstract

The spatial sensitivity of the human visual system depends on stimulus color: achromatic gratings can be resolved at relatively high spatial frequencies while sensitivity to isoluminant color contrast tends to be more low-pass. Models of early spatial vision often assume that the receptive field size of pattern-sensitive neurons is correlated with their spatial frequency sensitivity - larger receptive fields are typically associated with lower optimal spatial frequency. A strong prediction of this model is that neurons coding isoluminant chromatic patterns should have, on average, a larger receptive field size than neurons sensitive to achromatic patterns. Here, we test this assumption using functional magnetic resonance imaging (fMRI). We show that while spatial frequency sensitivity depends on chromaticity in the manner predicted by behavioral measurements, population receptive field (pRF) size measurements show no such dependency. At any given eccentricity, the mean pRF size for neuronal populations driven by luminance, opponent red/green and S-cone isolating contrast, are identical. Changes in pRF size (for example, an increase with eccentricity and visual area hierarchy) are also identical across the three chromatic conditions. These results suggest that fMRI measurements of receptive field size and spatial resolution can be decoupled under some circumstances - potentially reflecting a fundamental dissociation between these parameters at the level of neuronal populations.

## Highlights

• Novel use of fMRI population receptive field (pRF) mapping, using chromatic stimuli.
• Spatial frequency sensitivity in early visual areas measured with fMRI.
• Differences in spatial sensitivity found between S-cone and luminance conditions.
• No significant differences in pRF sizes between S-cone and luminance conditions.
• Suggests that pRF sizes and spatial resolution are not coupled.

## Introduction

The three pathways that contribute to human color vision originate in different retinal combinations of the signals from the long, medium and short-wave sensitive cone photoreceptors (Hurvich and Jameson, 1957, Jameson and Hurvich, 1968). One pathway processes achromatic luminance (L + M), and two are isoluminant chromatic pathways: ‘red vs. green’ (L-M) and ‘yellow vs. blue’ (S-cone isolating). These precortical physiological pathways can also be probed by psychophysical experiments, which demonstrate differences in their spatial frequency tuning profiles (Johnson et al., 2010, Kim et al., 2013, Mullen, 1985, Owsley et al., 1983, Poirson and Wandell, 1993, Poirson and Wandell, 1996, Webster et al., 1990). For luminance stimuli, these profiles are band-pass (peak sensitivity ∼4 cycles per degree (cpd)), whereas isoluminant chromatic stimuli functions are typically low-pass (peak sensitivity <1 cpd) (Webster et al., 1990).

The nature of simple *pre-cortical* center/surround receptive field structures means that receptive field size and preferred spatial frequency are correlated. Specifically, linear simple cells with large receptive fields respond to low spatial frequencies and *vice versa* (Chen et al., 2009, Cleland et al., 1979, Enroth-Cugell and Freeman, 1987, Irvin et al., 1993). However, this relationship ultimately breaks down in visual cortex. For instance, neurons have very large receptive fields in higher visual areas but can, nevertheless, be driven by stimuli with high spatial frequency content (Rajimehr et al., 2011). This non-linearity is typical of complex cells, in which spatial tuning is independent of receptive field sizes (Movshon et al., 1978).

Functional Magnetic Resonance Imaging (fMRI) allows us to record the average response from groups of neurons inside individual voxels that measure on the order of a cubic millimeter. By fitting the responses of these neuronal populations to simple high contrast achromatic stimuli, Dumoulin and Wandell (2008) showed that it is possible to estimate the population receptive field (pRF) size as well as preferred spatial field location for each voxel in visual cortex.

The stimuli used in pRF mapping must activate a subset of all the neurons in each voxel. For example, if the stimuli contain only very low spatial frequencies, they are unlikely to drive responses in neurons tuned to fine details. Similarly, isoluminant stimuli cannot drive neurons that respond only to achromatic contrast. Several groups have performed experiments altering the pRF mapping stimuli along some spatial dimensions (e.g. logarithmically-scaled bar widths, second order orientation, hybrid rings/wedges, and multifocal arc stimuli) and have reported effects on both the quality and the parameters of the resulting pRF models (Alvarez et al., 2015, Binda et al., 2013, Yildirim et al., 2017). However, all groups to date have used black and white 100% contrast carrier patterns. The pRF estimates produced are therefore all necessarily driven by neurons responding to high-contrast achromatic stimuli.

Here, we asked whether pRF size estimates change as a function of stimulus chromaticity. Because the spatial frequency sensitivity of isoluminant chromatic pathways is much lower than that of the achromatic pathway, we hypothesized that pRFs measured using isoluminant stimuli would be, on average, larger than those measured using achromatic stimuli - particularly for the S-cone condition, as the sparse density of S-cones in the retina limit the spatial resolution of the S-cone pathway (Williams et al., 1993). This predicted outcome is partly dependent on the type of cells contributing to the population; the hypothesis assumes that we are able to primarily record the activity of populations of linear simple cells within each voxel. However, studies of cat and monkey striate cortex indicate an approximately even split of cells classified into linear and complex cell types (Skottun et al., 1991) while recent work suggests that the distinction between simple and complex cells may be, in part, a function of spiking nonlinearities (Mechler and Ringach, 2002, Priebe et al., 2004) and so responses from both cell classes may be indistinguishable when measured by fMRI which is predominantly sensitive to presynaptic changes in membrane potential (Logothetis, 2000).

Therefore, an alternate hypothesis, that accounts for a larger contribution from complex cells, would not predict a difference in pRF sizes between conditions because these cells, as described above, demonstrate little correlation between receptive field size and spatial frequency tuning.

We performed two fMRI experiments, and a psychophysical experiment. One fMRI experiment confirmed that we could measure fMRI correlates of variations in spatial sensitivity in the different color channels, using full-field gratings of different spatial frequencies that stimulated each of the three axes from a cone excitation color space (the Macleod-Boynton color space) (MacLeod and Boynton, 1979). We performed the psychophysical experiment to measure contrast sensitivity functions for each of the conditions, using spatial 2-alternative-forced-choice (2AFC) tasks. The resulting functions agreed with previous behavioral measurements of spatial frequency tuning between these channels. Finally, we conducted pRF mapping with fMRI using spatially broadband carriers that stimulated the same, isolated axes in Macleod-Boynton color space, to ask whether we could measure systematic effects of chromaticity on pRF sizes across the visual field. Although we measured robust pRFs at all eccentricities and the size of these pRFs changed consistently with both eccentricity and visual area (in line with previous studies), no significant effects of chromaticity were observed in the pRF data. We discuss these findings in relation to early work on complex cell receptive field size and spatial frequency tuning.

## Methods

### Subjects

Six color-normal trichromats (two female) with a mean age of 28.7 years (±8.1 years) were recruited for this study. All subjects had previously taken part in retinotopic fMRI scans, and had normal or corrected-to-normal vision. Five of these subjects took part in the spatial sensitivity fMRI and psychophysical studies (two female, mean age 28 years (±8.9 years)), and all six subjects took part in the pRF study. The ethics committees at the York Neuroimaging Centre and the Department of Psychology at the University of York approved these experiments.

### Experiment and stimulus design

The stimuli used in these experiments were designed and presented using Psykinematix software (KyberVision, Montreal, Canada: psykinematix.com) on an Apple Mac computer (Apple computers, USA). The delivery system used for the visual stimulus in the scanner was an Epson EB-G5900 projector with a long throw lens, which projected the stimulus onto a custom-made acrylic screen. The participant viewed the screen with a mirror set-up in the scanner. For the psychophysical tasks, the subjects viewed the stimulus on a NEC MultiSync 200 CRT monitor, running at 100Hz. For both displays, gamma correction was performed using a ‘Spyder4’ (Datacolor, NJ, USA) display calibrator. The same calibrator was used to measure the color properties of the RGB guns on the CRT monitor, whereas spectral measurements of the scanner screen RGB channels were made through the viewing mirror using a ‘Jaz’ (Ocean Optics, FL) photospectrometer at 2 nm resolution and imported into Psykinematix.

#### Isoluminance

To ensure the chromatic stimuli were isoluminant for each subject, minimum motion isoluminance tasks were carried out while inside the scanner, so that the stimuli could be specifically tailored for each subject's isoluminant point. Subjects fixated centrally while adjusting the color of a drifting grating that was placed in their lower left periphery. The grating had a 2° radius, centered at an eccentricity of 7° from the fixation point, with a drift rate of 1°/s and spatial frequency of 1 cpd. The point at which the drifting motion was minimized was chosen to reflect the isoluminant point of the stimulus (Anstis and Cavanagh, 1983). The color direction of the grating was specified within the Psykinematix software using LMS values in MacLeod-Boynton color space (MacLeod and Boynton, 1979), using the 2° cone fundamentals from Stockman and Sharpe (2000). The RMS (root mean square) contrasts used for the stimuli in the isoluminance tasks matched those used in the spatial sensitivity and pRF fMRI experiments for the same conditions: L-M = 4%, S-cone = 15%. These contrast values approximately equalize responses in primary visual cortex (Kane et al., 2011), and were calculated as 3× the average contrast detection levels measured using a spatial 4-alternative-forced-choice (4AFC) method, with circular (2° diameter) white noise stimuli placed at 7° eccentricity from the central fixation mark; the luminance contrast threshold acquired with the same method yielded a 5% contrast.

Three repeats of the isoluminance adjustments were made, and the average of these values was used. Subjects practiced these minimum motion tasks outside the scanner in the laboratory (on the calibrated CRT monitor) prior to performing them in the scanner. In all cases, isoluminant directions were very close to those predicted by the nominal MacLeod-Boynton axes.

#### Spatial sensitivity stimuli

**Fig. 1:**
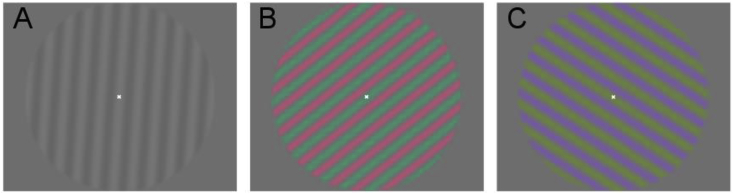
Example stimuli for the spatial sensitivity fMRI experiment. (A) luminance, (B) L-M, and (C) S-cone isolating conditions, at a spatial frequency of 0.5 cpd. Isoluminant directions were determined separately for each observer using a minimum motion paradigm.

The stimuli used in the spatial sensitivity experiment were sinusoidal gratings presented within a circular window (radius 10°) with one of three spatial frequencies (0.5, 2 and 8 cpd). Orientation was randomized and contrast polarity was reversed at a temporal frequency of 2Hz (see examples in Fig. 1). For all subjects, the RMS cone contrast levels of the Luminance, L-M and S-cone stimuli were set to 5%, 4% and 15% respectively, as used in the isoluminance tasks described above.

Subjects fixated centrally throughout, and performed a demanding attentional task (button press when the fixation cross changed) that was not locked to the timing of the grating.

An event-related design was used to present the stimuli from each event condition; there were a total of 10 events (3 spatial frequencies for 3 conditions, plus one blank condition). Each event was presented for 3 s (1 TR) with a randomized inter-stimulus interval length of between 3 and 6.5 s. Each event was presented four times in a complete scan, with all events presented in a randomized order. A total of four scans were completed for each subject, which resulted in 16 trials for each event condition.

#### Psychophysical stimuli

Contrast detection thresholds were measured for each color condition (luminance, L-M, and S-cone isolating) at each of the spatial frequencies used in the spatial sensitivity fMRI experiment. A spatial 2AFC method was used with a Bayesian staircase procedure (Kontsevich and Tyler, 1999) to obtain 75% correct detection thresholds. The task was performed at two eccentricities: 2° and 8° (horizontally from fixation to the center of each grating). Thresholds were determined for each condition in separate blocks of trials.

Stimuli were circularly-windowed sine-wave gratings (2° diameter), and trial locations were outlined with thin white circles to remove spatial uncertainty. A total of 200 trials (presented for 100 ms) were carried out for each eccentricity and spatial frequency combination plus 10 practice trials of each, which were not included in the analysis.

For each of the chromatic conditions, minimum motion tasks similar to those described for the fMRI experiments were carried out first to set the isoluminance levels for each eccentricity and observer.

#### pRF stimuli

The stimuli for each condition in the pRF experiment matched the spatial sensitivity stimuli in contrast, isoluminance values used, total eccentricity (20° diameter), and temporal frequency (2Hz).

**Fig. 2:**
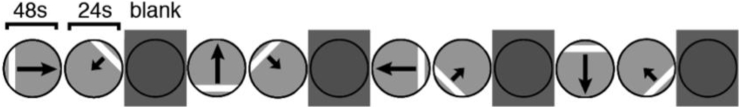
Schematic of the bar movement throughout a single pRF scan. The ‘blank’ dark gray sections represent the mean-luminance periods (24 s). Larger arrows indicate that the bar swept across the full length of the direction (48 s), smaller arrows indicate that the bar swept across half of the direction (24 s).

A bar stimulus similar in general form to those described in other experiments (Alvarez et al., 2015, Binda et al., 2013, Dumoulin and Wandell, 2008) was used; a single bar (width 0.5°) within a circular aperture (10° radius) moved in one of eight directions with each ‘sweep’ across the field lasting 48 s. Four periods of mean luminance were included to provide a baseline condition within each scan, these periods always occurred in the second half of diagonal bar sweeps and lasted 24 s (see Fig. 2). Subjects carried out a maximum of four scans of each condition over two or three sessions.

**Fig. 3:**
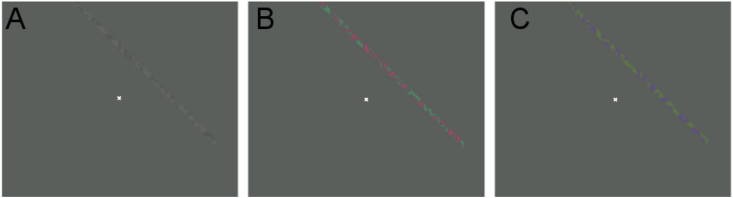
Examples of the stimuli from the pRF experiment. Shown for each condition (with contrast (%)): (A) Luminance (5%), (B) L-M (4%), and (C) S-cone isolating (15%).

To equalize spatial frequency power across the spectrum, carriers consisted of a white noise pattern that updated at 2Hz; examples of the pRF stimuli used in each condition can be seen in Fig. 3. In Appendix A, we illustrate the representation of this stimulus in the Fourier domain: the windowed white noise generates a smooth spread of power in the spatial frequency domain. The effect of increasing bar width is to slightly increase the overall power of the stimulus in Fourier space but it has no effect on the relative distribution of frequency components.

To help the subjects maintain central fixation, the same attentional task from the spatial sensitivity experiment was used.

### MRI protocol

fMRI scans were carried out using a GE 3 T HDx Excite MRI scanner, with a 16-channel posterior surface coil (Nova Medical, Wilmington, MA) covering the occipital pole. The subject's head was positioned in the coil mount and surrounded by foam padding and a forehead strap to ensure the head was stable and that the subject was comfortable. Scan slices were aligned to cover the region containing and surrounding the calcarine sulcus (the anatomical region containing the primary visual cortex). A total of 39 EPI slices were taken within an FOV of 192 × 192 mm^2^, with 2 mm^3^ isotropic voxels (TR = 3000 ms, TE = 30 ms, flip angle = 90°, acquisition/reconstruction matrix = 96 × 96). Four ‘dummy’ TRs (12 s) were included at the beginning of each scan to allow the signal to reach magnetic equilibrium.

In addition to the functional scans, a proton density (PD) scan with the same spatial prescription as the EPI data was acquired at the beginning of each session – this scan was used to align the fMRI data to a high-resolution (1 x 1 x 1mm) T1-weighted structural scan of the full brain acquired prior to the fMRI sessions in the same GE 3 T HDx Excite MRI scanner, using an 8-channel surface coil to minimize magnetic field inhomogeneity.

### Data processing

All functional data processing was performed using the 2015 version of the VISTA software (https://web.stanford.edu/group/vista/cgi-bin/wiki/index.php/Software) (Vista Lab, Stanford University), running under MATLAB 2012a (The MathWorks Inc., Natick, MA, USA). fMRI scan data were imported and motion corrected between and within scans from each session using a maximum likelihood alignment routine (Nestares and Heeger, 2000).

T1-weighted high resolution scans were used to reconstruct a structural model of each subject's brain using a combination of FSL (http://fsl.fmrib.ox.ac.uk/fsl/fslwiki/) (Smith et al., 2004), Freesurfer (http://surfer.nmr.mgh.harvard.edu/) (Dale et al., 1999, Reuter et al., 2012) and the VISTA software. The functional scans were aligned to the anatomical structural image using the PD scan acquired at the beginning of the functional scan session. Alignments were checked for accuracy by visual inspection and minor adjustments were made using manually-placed control points.

After the functional scans from each session were aligned to these high-resolution anatomies, analyses were confined to the segmented cortical gray matter sheet (Wandell et al., 2000).

Regions of interest (ROIs) of early visual areas V1 through to V4 were identified using the retinotopic output of the pRF modelling (described below). Further ROIs were created within each visual area, to produce two eccentricity group levels - foveal (<2° visual angle) and peripheral (between 8° and 10° visual angle). The same ROIs were used to analyze both the spatial sensitivity data and the pRF data.

#### pRF experiment – data processing

pRF sizes and positions were estimated for each voxel and chromaticity condition using the standard pRF modelling algorithm described by Dumoulin and Wandell (2008) and implemented with the 2015 VISTA software tools. Modelling was performed on time series data averaged across all repetitions of the same chromaticity condition using a standard ‘difference of gammas’ hemodynamic response function (HRF) from the SPM analysis package (Friston et al., 2006). The final pRF estimates only include voxels that have at least 10% of the variance explained by the model fit.

**Fig. 4:**
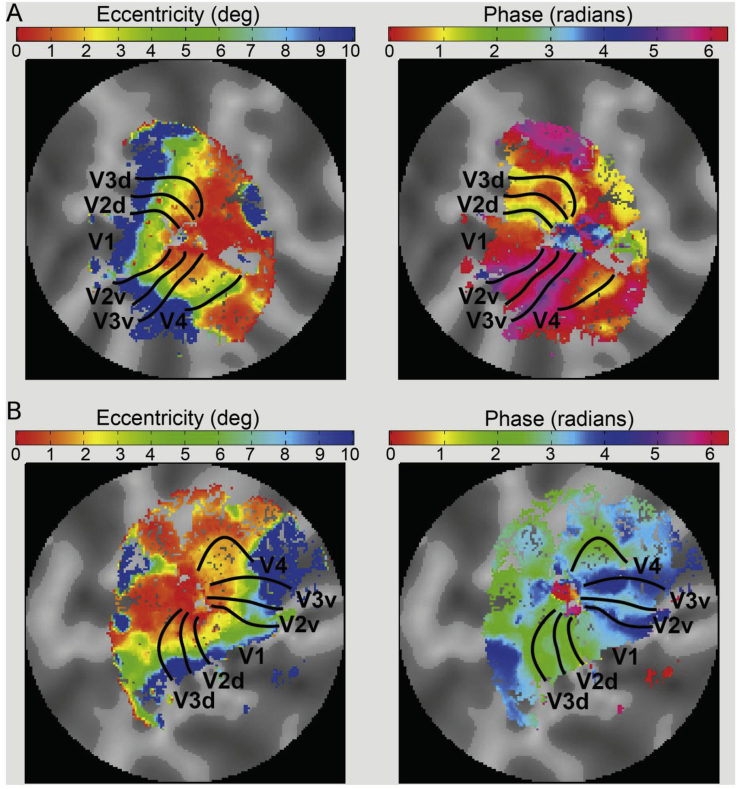
Retinotopic maps for one subject. Eccentricity (left) and polar angle (right) phase maps are shown, which were used to identify visual area ROIs in the left (A) and right (B) hemispheres. Boundaries of the visual areas are overlaid on the maps.

**Fig. 5:**
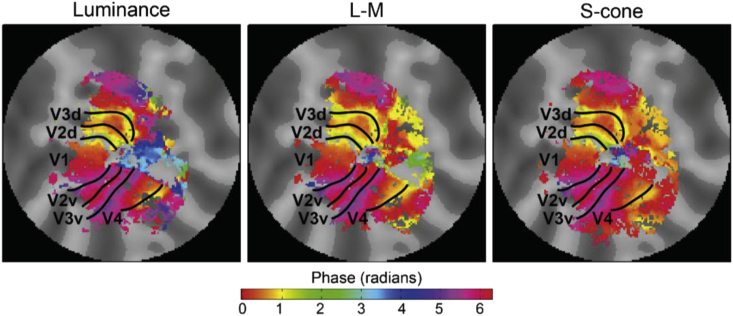
Left hemisphere retinotopic phase maps for one subject. Shown for each of the conditions separately (luminance, L-M, S-cone). The boundary lines overlaid in black are positioned in the exact same locations for each condition, and in the same locations as those shown in Fig. 4A (for the same subject demonstrated here).

We also applied pRF modelling to a grand average of all scans (i.e. across all chromatic and luminance conditions); the retinotopic eccentricities and polar angles from these grand averages were used to estimate the boundaries of the early visual areas (V1-V4). These regions of interest (ROIs), marking each individual's early visual areas, were drawn by hand on a flattened representation of the cortical surface and checked by at least one other expert observer, see Fig. 4 for an example from one subject. Retinotopic maps produced by each chromatic condition show essentially identical visual area boundaries (as shown in Fig. 5, for the same subject), and therefore the same grand average ROIs were used for all conditions within each subject. The foveal and peripheral eccentricity ROIs were created by restricting the data within each visual area for the desired visual angles, using the eccentricity values from the model.

#### Spatial sensitivity experiment – data processing

Event-related data were analyzed using a general linear model (GLM) within the VISTA software tools. The beta weights for each condition were extracted for each subject for each event, and group averages were produced for the ROIs described above.

For each color condition, the responses to the 8 cpd spatial frequency stimuli were particularly low and subjects reported that these stimuli were very hard to see – possibly due to limitations in the resolution of the scanner stimulus display. Therefore, only the 0.5 and 2 cpd conditions were used to measure spatial sensitivity. The difference in signal response between these two conditions was calculated to produce a single value, referred to here as spatial sensitivity. Negative values indicated a greater sensitivity to lower spatial frequencies, whereas positive values indicated greater sensitivity to higher spatial frequencies. For each subject, and within each ROI for each condition, the 2 cpd and 0.5 cpd beta values were first normalized to the peak response out of the two spatial frequencies and then the difference between the normalized values was calculated (see Equation (1)).

Equation (1) Spatial sensitivity calculation. peakVal_cond_ is the max value out of both 2cpdVal_cond_ and 0.5cpdVal_cond_, and _cond_ refers to the particular condition, i.e. one of the chromaticity groups (luminance, L-M, or S-cone) for a particular ROI.

(1) $$SS_{cond}=\;\left(\frac{2cpdVal_{cond}}{peakVal_{cond}}\right)-\left(\frac{0.5cpdVal_{cond}}{peakVal_{cond}}\right)$$

## Results

### Psychophysical contrast sensitivity functions

**Fig. 6:**
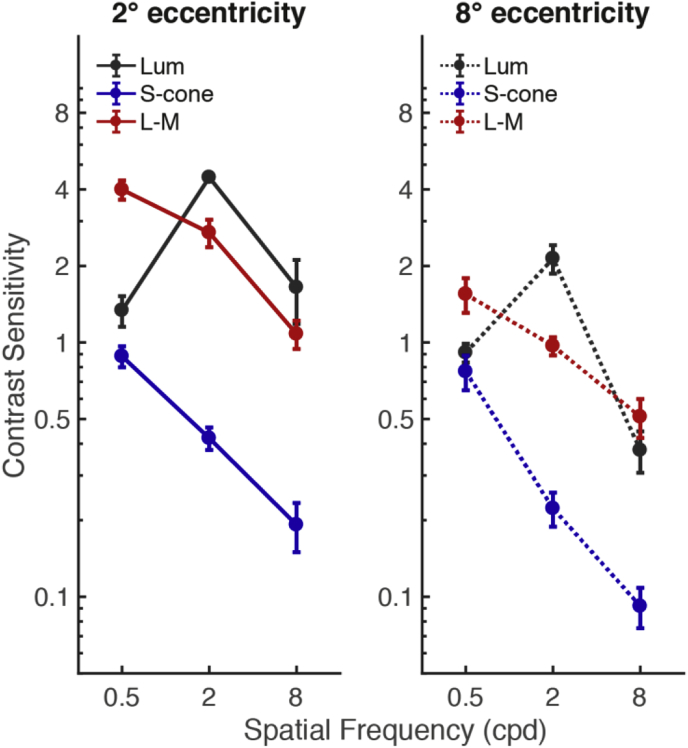
Mean contrast sensitivity functions. Contrast sensitivity (reciprocal of contrast detection thresholds (%)) across subjects (n = 5) plotted as a function of spatial frequency, with standard error bars. Shown for each condition (luminance (gray markers), L-M (red markers) and S-cone (blue markers)) at two eccentricities (2° (solid lines) and 8° (dashed lines)).

Fig. 6 shows the psychophysical contrast sensitivity functions for each condition (Luminance, L-M, and S-cone isolating) at two eccentricities (2° and 8°); values are the means across subjects with standard error bars. For both eccentricities, the chromatic conditions show low-pass sensitivity functions, whereas the luminance condition shows peak sensitivity at the middle spatial frequency value (2 cpd). Between eccentricities, lower contrast sensitivities are observed as a function of eccentricity, with the L-M condition showing the greatest overall difference between the 2° and 8° conditions. Factors of eccentricity, condition, and spatial frequency were entered into a repeated-measures ANOVA to identify the effect of each on the contrast sensitivity values; significant main effects were found for each factor (eccentricity (*F*(1,4) = 179.921, *p* = 10^−4^), condition (*F*(2,8) = 78.730, *p* = 10^−6^), and spatial frequency (*F*(2,8) = 153.965, *p* = 10^−6^)).

Each of these observations have been reported by many other groups (Mullen, 1985, Mullen and Kingdom, 2002, Rovamo et al., 1999, Webster et al., 1990), and, in particular, they support the assertion that psychophysically-defined S-cone isolating pathways have low spatial sensitivity. They also serve as a useful validation of our stimulus generation and presentation pathway: significant errors in, for example, our calibration procedures would have led to luminance contamination of our nominally-isoluminant stimuli and a corresponding increase in similarity between the luminance and isoluminant spatial sensitivity functions.

### Statistical analysis

#### Spatial sensitivity

**Fig. 7:**
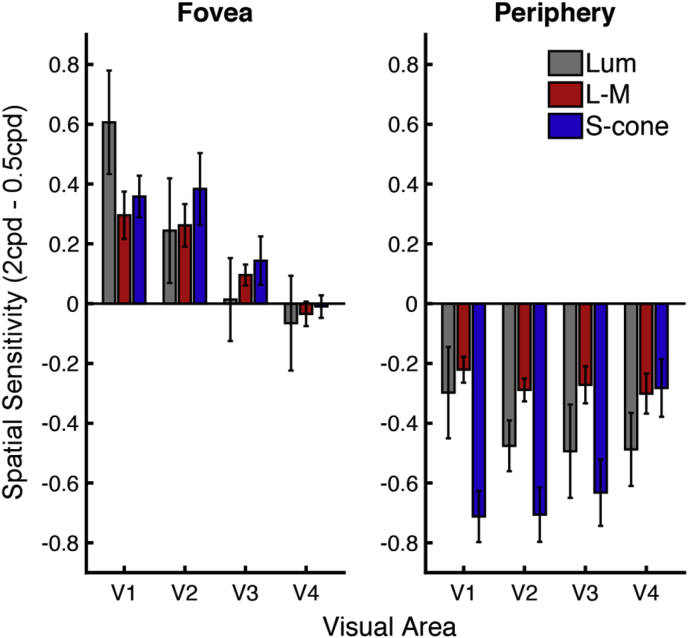
Mean spatial sensitivity values for foveal and peripheral eccentricities. Mean across subjects (n = 5), with error bars showing the standard error of the means. Values for each condition (luminance (gray bars), L-M (red bars) and S-cone (blue bars)) are shown across visual areas for foveal (left) and peripheral (right) eccentricity ROIs.

The spatial sensitivity data were analyzed using the foveal and peripheral eccentricity ROIs for each visual area, these data are plotted in Fig. 7. A repeated-measures ANOVA was carried out using factors of visual area, eccentricity and condition, to determine any effect on spatial sensitivity (the difference between responses to the 2 and 0.5 cpd spatial frequencies).

Mauchly's test of Sphericity was violated only for the interaction between eccentricity and visual area (χ^2^(5) = 12.243, *p* = 0.042), therefore a Greenhouse-Geisser correction was applied to the results of this interaction. There was a significant effect of eccentricity (*F*(1,4) = 78.636, *p* = 0.001), and visual area (*F*(3,12) = 11.110, *p* = 0.001), but no significant main effect of condition (*F*(2,8) = 0.655, *p* = 0.545). However, *all* interactions were shown to be significant: eccentricity and visual area (*F*(1.682,6.726) = 8.375, *p* = 0.017, Greenhouse-Geisser corrected), eccentricity and condition (*F*(2,8) = 4.682, *p* = 0.045), condition and visual area (*F*(6,24) = 6.330, *p* = 0.0004), and the interaction between all factors (*F*(6,24) = 3.805, *p* = 0.008). Visual observation of the data indicate that the significant interactions may primarily be driven by the peripheral S-cone condition. To explore this further, paired comparisons were made between each of the conditions at each eccentricity, within area V1; paired t-tests were carried out between the conditions within each eccentricity (i.e. six comparisons, reducing the significance criteria with Bonferroni correction to 0.008). The S-cone condition did significantly differ from both the L-M condition (*t*(4) = −8.002, *p* = 0.001) and the luminance condition (*t*(4) = −5.793, *p* = 0.004), within the peripheral eccentricity. No other condition pairs reached significance.

#### pRF mapping

**Fig. 8:**
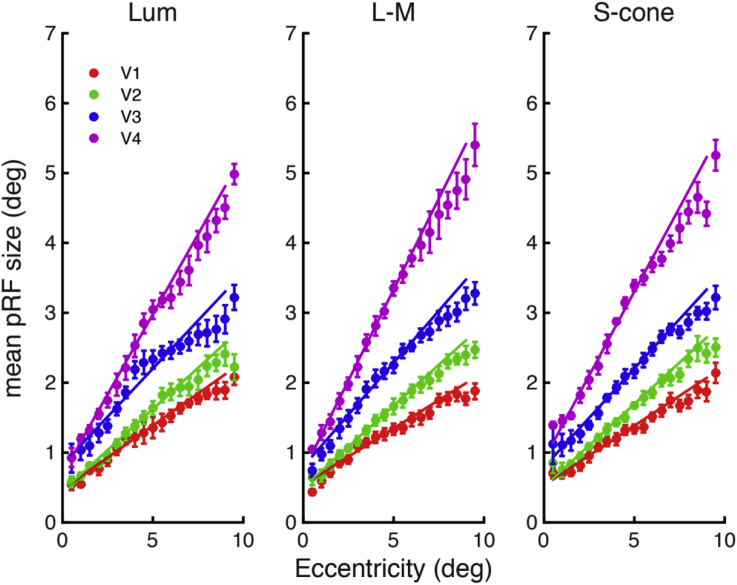
pRF sizes plotted as a function of eccentricity. Mean pRF sizes across subjects (n = 6), with standard error bars, are shown across eccentricities for each visual area (V1 (red), V2 (green), V3 (blue) and V4 (magenta)), with line of best fit shown for each visual area. Individual plots are provided for each condition (from left to right: luminance, L-M, and S-cone).

pRF sizes for each chromatic condition are plotted as a function of eccentricity in Fig. 8, and are separated by visual area (see figure legend). For all conditions, increases in pRF sizes can be observed as a function of both eccentricity and visual area.

**Fig. 9:**
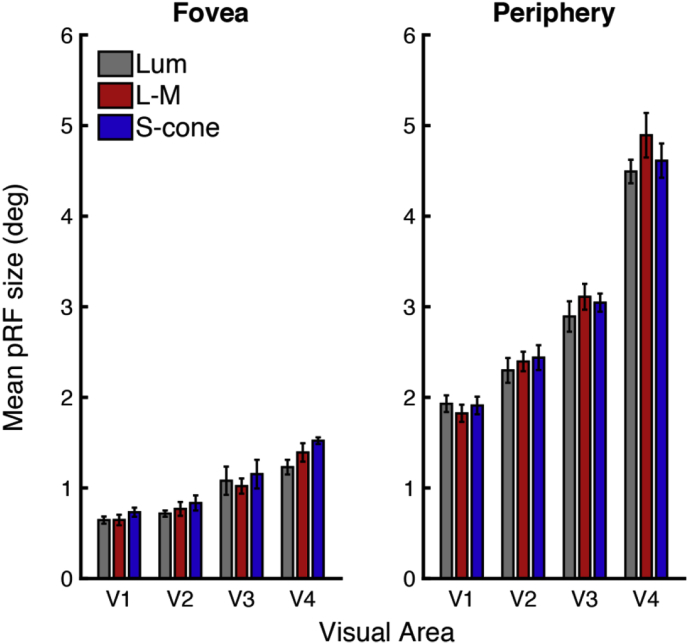
Mean pRF sizes for foveal and peripheral eccentricities. Mean pRF sizes across subjects (n = 6), with standard error bars, are shown for each condition (luminance (gray bars), L-M (red bars) and S-cone (blue bars)), with bars grouped by visual area. Plots are split by eccentricity: foveal (left) and peripheral (right).

As with the spatial sensitivity data, the data were analyzed using the foveal and peripheral eccentricity ROIs for each visual area, plotted in Fig. 9, using a repeated-measures ANOVA with factors of visual area, eccentricity and condition. Mauchly's test of Sphericity was violated for the visual areas factor (χ^2^(5) = 15.695, *p* = 0.010), and therefore a Greenhouse-Geisser correction was applied prior to the interpretation of the visual area factor and associated interactions where Mauchly's test could not be run (i.e. three-way interaction and the interaction with condition).

In line with the observations made from the plotted data in Fig. 8, highly significant effects were observed for the factors of eccentricity (*F*(1,5) = 2458.257, *p* = 10^−7^), and visual area (*F*(1.574,7.871) = 107.981, *p* = 10^−5^, Greenhouse-Geisser corrected), and a significant interaction was found between these two factors (*F*(3,15) = 85.102, *p* = 10^−8^). However, no significant effect of condition was observed (*F*(2,10) = 2.37, *p* = 0.144), and no significant interactions with the condition factor were found: condition and visual area (*F*(2.580,12.899) = 1.253, *p* = 0.327, Greenhouse-Geisser corrected), condition and eccentricity (*F*(2,10) = 1.905, *p* = 0.199), or for the three-way interaction between all factors (*F*(1.765,8.825) = 1.675, *p* = 0.241, Greenhouse-Geisser corrected).

**Fig. 10:**
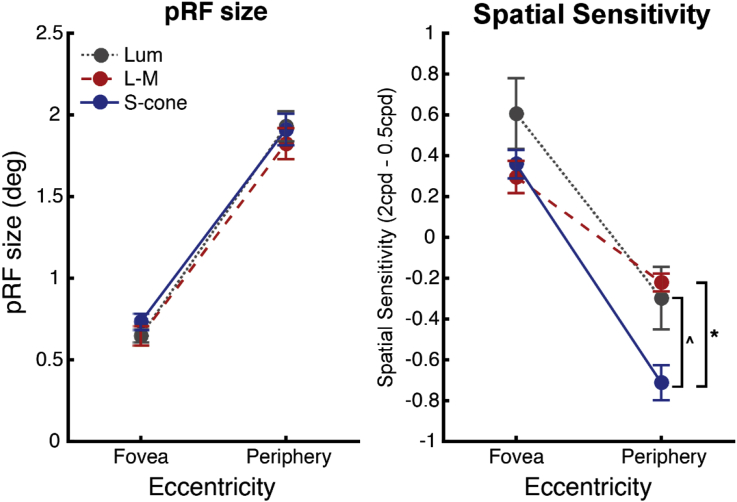
Data from V1: Mean pRF sizes (degrees) and spatial sensitivity values (2cpd - 0.5cpd) plotted as a function of eccentricity. Data are shown for each condition (luminance (gray markers), L-M (red markers) and S-cone (blue markers)), from visual area V1. Mean pRF sizes (n = 6) and mean spatial sensitivity values (n = 5) both shown with standard error bars. Significant results of paired t-tests between the peripheral spatial sensitivity indices are indicated: ˆp = 0.024, *p = 0.006 (Bonferroni corrected), see text in Statistical analysis section ‘Spatial sensitivity’for details.

In contrast to the significant paired comparisons shown for the spatial sensitivity data in area V1 (see Statistical analysis section ‘Spatial sensitivity’), for the pRF data no significant differences were found between any of the condition pairs at either the foveal or peripheral eccentricities. Both sets of V1 data are shown side-by-side in Fig. 10.

We also implemented a Compressive Spatial Summation (CSS) pRF model (Kay et al., 2013) on our data. This has been shown to account for more of the variance in the fMRI signal than the original linear pRF model (Dumoulin and Wandell, 2008). However, in the visual areas we analyzed (V1-V4) we did not find a significant difference in the amount of variance explained within each condition by the CSS model compared to the original pRF model (using paired t-tests, and Bonferroni correction). Furthermore, this model did not produce any differences in the pRF size estimates (the results of a repeated-measures ANOVA found the same overall effects and interactions as reported above). The detailed output of this model is reported in Appendix B.

It is possible that between voxels there is variability in the number of neurons preferentially responsive to each condition, which may bias the mean pRF size produced for each voxel. Therefore, in order to control for any such bias, we repeated the analysis using different voxels for each condition, namely those that preferentially responded to each condition, as determined by the amount of variance explained by each of the conditions. For instance, the voxels that were used for the luminance condition in this analysis were those that had a greater amount of the variance explained than either the L-M or S-cone conditions. A repeated-measures ANOVA showed no main effect of condition (*F*(2,10) = 2.335, *p* = 0.147), while there were significant main effects of eccentricity (*F*(1,5) = 311.016, *p* = 10^−4^) and visual area (*F*(3,15) = 51.741, *p* = 10^−7^), and a significant interaction between these two factors (*F*(3,15) = 42.875, *p* = 10^−6^). In this analysis, a significant interaction was also observed between condition and eccentricity (*F*(2,10) = 11.963, *p* = 0.002), however, the three-way interaction between all factors remained insignificant (*F*(6,30) = 1.165, *p* = 0.351), as was the interaction between condition and visual area (*F*(6,30) = 1.442, *p* = 0.232).

Paired comparisons between conditions for foveal and peripheral eccentricities within V1 showed no significant differences between any of the pairings, in agreement with the original analysis.

## Discussion

We measured pRF sizes using isoluminant chromatic stimuli that confine responses to a limited set of pre-cortical pathways – an experiment suggested by Dumoulin and Wandell (2008) in the first paper to introduce this fMRI technique. We asked whether cortical neuronal populations tuned to isoluminant color directions have larger average receptive field sizes than those driven predominantly by achromatic patterns. We used fMRI to measure pRF sizes and spatial frequency sensitivity across eccentricities and visual areas (V1 to V4) and found no effect of chromatic condition on pRF sizes: pRF sizes increased systematically across all conditions, with an identical increase in size as a function of both eccentricity and visual area. Conversely, we did observe differences in spatial frequency sensitivity between conditions. Specifically, the S-cone isolating condition showed a significantly greater sensitivity to lower spatial frequencies than either the luminance or L-M conditions in the more peripheral location.

Typical contrast sensitivity functions (CSFs), acquired behaviorally, show that S-cone isolating stimuli produce low-pass CSFs, and an achromatic luminance stimulus produces a band-pass CSF, which peaks in sensitivity at approximately 4 cpd in the fovea (Mullen, 1985, Webster et al., 1990). We also replicated these findings here, at both 2° and 8° eccentricities, using the same subjects from the fMRI experiments. In our fMRI spatial sensitivity experiment, all conditions showed greater sensitivity to higher spatial frequencies in the fovea (2 cpd > 0.5 cpd), and greater sensitivity to lower spatial frequencies in the periphery (0.5 cpd > 2 cpd). In the periphery, our data showed significantly greater low-pass-type responses for the S-cone isolating stimuli compared to *both* the luminance and the L-M conditions; the L-M condition did not significantly differ from the luminance condition in spatial sensitivity.

Psychophysical experiments are often used to estimate features of the underlying physiology. However, our psychophysical data do not match the fMRI data from our spatial sensitivity experiment directly. This may be due to several aspects of the stimuli that differ between the experiments. For instance, psychophysical studies often operate at detection or discrimination thresholds (low contrast differences and short stimulus durations), whereas stimuli in fMRI studies are presented at suprathreshold contrast levels for longer periods (∼3 s). These above-threshold contrasts may simply drive more neurons and broaden the population-level tuning curves compared to threshold level responses. Suprathreshold stimuli may also drive some neuronal populations to saturation, flattening population tuning curves and reducing the effective response differences between chromatic channels. We note, for example, that Poirson and Wandell (1993) estimate that the SF tuning of neuronal chromatic channels measured in suprathrehsold experiments may be far more similar than behavioral responses suggest because much of the high spatial frequency power is removed by optical factors. Another way of viewing this observation is that cortex may have relatively large populations of S-cone-driven cells sensitive to high spatial frequencies (and perhaps with relatively small RFs) but that these are invisible at low contrasts because axial chromatic blur will remove the stimulus features that drive them. At high contrast, there may be enough residual power at high spatial frequency to elicit a response from this population.

One way to address this issue would be to perform pRF mapping using threshold-level stimuli. Given sufficient SNR (signal to noise ratio) or a long-enough recording session, responses driven by threshold-level differences can be measured (Ress and Heeger, 2003), and it might, in principle, be possible to perform pRF mapping experiments in this manner, but this is beyond the scope of our study.

Nevertheless, our data are in broad agreement with recent fMRI studies of spatial frequency tuning. For instance, Henriksson et al. (2008) show that for achromatic stimuli the mean spatial frequency preferences of voxels decreased both with ascending visual area (from V1 to V3A) and with increasing eccentricity. Both of these effects are observed in our data. More recently, D'Souza et al. (2016) found the same decrease in spatial frequency tuning with increased eccentricity within V1, for both achromatic *and* chromatic (L-M and S-cone isolating) stimuli. In line with our findings, D'Souza et al. found that responses in foveal V1 demonstrated band-pass responses for all pathways, with the luminance stimuli producing smaller responses than the chromatic conditions for the lower spatial frequencies (our data showed a non-significant trend in this direction). Additionally, at a peripheral eccentricity of 9.8°, responses to S-cone stimuli decreased more rapidly with increasing spatial frequency than either the luminance or L-M conditions. This result is consistent with our finding that the relative spatial sensitivity in the S-cone condition was significantly lower than both luminance and L-M conditions at peripheral eccentricities in V1.

Despite these chromatically-driven differences in spatial sensitivity, we found no evidence of similar changes in pRF size. We hypothesized that, if the populations measured represented primarily linear simple cells, the pRF sizes would be significantly larger for chromatic stimuli – particularly for the S-cone condition – compared to achromatic, luminance stimuli. This hypothesis was based on the known differences in the spatial frequency tuning profiles of the pathways (described above), the retinal-level limitations of spatial resolution in the S-cone pathway, and negative correlations between receptive field sizes and preferred spatial frequency, recorded from retinal ganglion cells and single-cells within the lateral geniculate nucleus (LGN) of cats and primates (Cleland et al., 1979, Croner and Kaplan, 1995, Derrington and Lennie, 1984, Enroth-Cugell and Freeman, 1987, Irvin et al., 1993, Kremers and Weiss, 1997, O'Keefe et al., 1998; Xu et al., 2001). It was also informed by measurements of chromatic and achromatic neuronal receptive field sizes in both simple and complex cells, made using unit electrophysiology (Solomon et al., 2004), which suggest at least a two-fold difference in size between achromatic and S-cone receptive fields in V1. Our measurements of spatial sensitivity in V1, across all conditions in foveal and peripheral eccentricities, can also be used to make predictions about the magnitude of the expected differences in pRF sizes. Specifically, we used the linear line of best fit for pRF sizes vs. spatial sensitivity values, plotted for each of the eccentricity groups from the luminance condition, to predict pRF sizes in each eccentricity for the L-M and S-cone conditions, based on their spatial sensitivity values (‘*SS’*) from each eccentricity (‘*eccen’*): $pRF_{eccen}=\left(-1.418\times SS_{eccen}\right)+1.505$. Using this method, the S-cone condition is predicted to have larger pRF sizes than the luminance condition, with a difference of 0.34° in the fovea (larger by a factor of 1.55) and 0.58° in the periphery (larger by a factor of 1.3). The L-M condition is predicted to have a larger pRF size than the luminance condition in the fovea, with a difference of 0.43° (larger by a factor of 1.68), but a slightly smaller pRF size in the periphery, with a difference of −0.11° (smaller by a factor of 0.94).

The pRF technique does appear to reflect the receptive field sizes of the underlying neuronal population: previous fMRI pRF studies using achromatic stimuli (Alvarez et al., 2015, Binda et al., 2013, Dumoulin and Wandell, 2008) reported an increase in pRF sizes as a function of eccentricity, which agrees with other single-cell (Gattass et al., 1987, Van Essen et al., 1984) and 2-deoxyglucose uptake measurements (Tootell et al., 1988) and our spatial sensitivity data are surprisingly similar to those noted by earlier attempts to measure chromatic and achromatic population receptive fields using multiunit recordings (Victor et al., 1994). The increase in pRF size with eccentricity also mirrors the negative correlations reported between eccentricity and spatial frequency sensitivity (Cleland et al., 1979, Foster et al., 1985, Schiller et al., 1976, Troy, 1983). We consider two possible explanations for the apparent invariance in pRF sizes between chromatic and achromatic conditions in our data.

One possibility is that our pRF measurements may not be sensitive enough to detect differences between the chromatic stimulus conditions. However, this explanation seems unlikely, given the reliability of our data and the fact that our measurements are able to track the eccentricity-dependent changes in pRF sizes, which are consistent with changes in spatial frequency tuning across eccentricities seen for both luminance and isoluminant systems. Likewise, anticipated changes in pRF sizes are observed across visual areas. If the S-cone system had scaled pRF sizes consistently with spatial frequency sensitivity, we would expect to find a difference between pRF sizes for luminance and S-cone values; if we use our spatial sensitivity data to predict the pRF sizes (as described above), we could expect an increase of at least a factor of 1.3 in the S-cone condition. This change in pRF sizes should be clearly detectable given the sensitivity of our data.

Alternatively, there may be no difference in average pRF sizes between luminance and chromatic pathways in visual cortex. While the correlation between spatial frequency tuning and receptive field size is mandatory in *simple cell* receptive fields, early work on complex cell receptive field structure by Movshon et al. (1978), demonstrated that this relationship breaks down for complex cells: the spatial tuning of complex cells is independent of their receptive field sizes. As demonstrated in several fMRI studies, including our own, clear differences in spatial frequency tuning can be observed across eccentricities, visual areas, and between achromatic and chromatic pathways (D'Souza et al., 2016, Henriksson et al., 2008). However, these differences in spatial sensitivity need not be coupled with receptive field sizes if they reflect responses dominated by complex cells – perhaps because our stimuli could, potentially, drive second order contrast detection mechanisms, which have recently been shown to support pRF mapping in early visual areas (Yildirim et al., 2017), as well as first-order luminance contrast detectors. It is also possible that, for some reason, complex cells contribute proportionately more fMRI signal, and therefore mask sub-populations of linear simple cells that are tuned to spatial frequency as a function of receptive field size.

We also note that the spatial frequency/size prediction for L-M isoluminant stimuli is far less clear. Many so-called color-luminance cells respond to both L-M and Luminance contrast and most of these are sensitive to spatial structure (Johnson et al., 2004). Although our behavioral data suggest reduced spatial frequency sensitivity in the neurons tuned to L-M contrast at threshold, once our stimulus contrast was increased to generate a reliable BOLD response, it is possible that we stimulated a population of color-luminance neurons that may have responded to both L-M and achromatic contrast in a similar manner. Likewise, it has been shown that some neurons within V1 also represent combinations of the S-cone pathway with both L-M and luminance pathways (De Valois et al, 2000), and therefore responses from these cells may also be similar across all conditions.

To summarize, we used the same stimulus parameters (contrast, isoluminance, temporal frequency) to measure spatial frequency sensitivity and pRF sizes of neurons driven by the luminance, L-M and S-cone isolating pathways. Effects of chromatic condition were observed for the spatial sensitivity manipulation, with S-cone isolating stimuli producing significantly lower spatial sensitivity indices than either the luminance or L-M conditions in the peripheral areas of V1. No effects of chromaticity were observed in the pRF data. We conclude that the invariance observed in pRF measurements was a result of an actual invariance in population-average receptive field sizes between these pathways. We suggest that this may be due to the prevalence of color-luminance cells as well as the presence of complex, pattern-sensitive cells in the visual cortex, which do not demonstrate a linear relationship between receptive field size and spatial sensitivity.

## Figures and Tables

**Fig. A.1:**
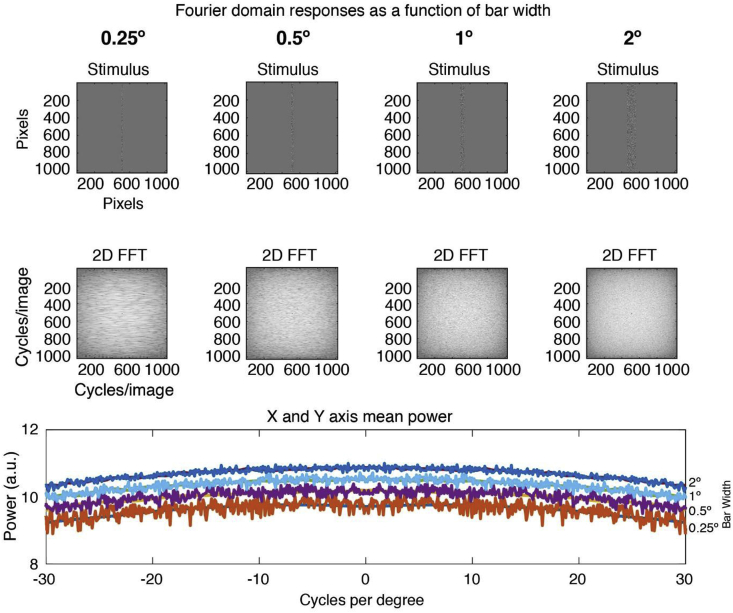
Fourier domain representations of pRF stimuli with different bar widths (0.25, 0.5, 1 and 2 degrees of visual angle). Top row: A representative stimulus bar extending ±5° about fixation filled with white noise and with a sigma = 0.01° spatial filter applied to mimic blurring due to the projection system. Middle row: 2D log(abs(FT)) of the input stimulus. Bottom row: Mean projected log power along Y axis and X axis. The projections for any individual bar width have an identical overall profile with the only difference being that the Y axis projections are noisier and thus tend to hide the X axis projections. Increasing the bar width increases the overall power in the stimulus (the projected power plots go up with bar width) but there is no change in preferred spatial frequency.

**Fig. B.1:**
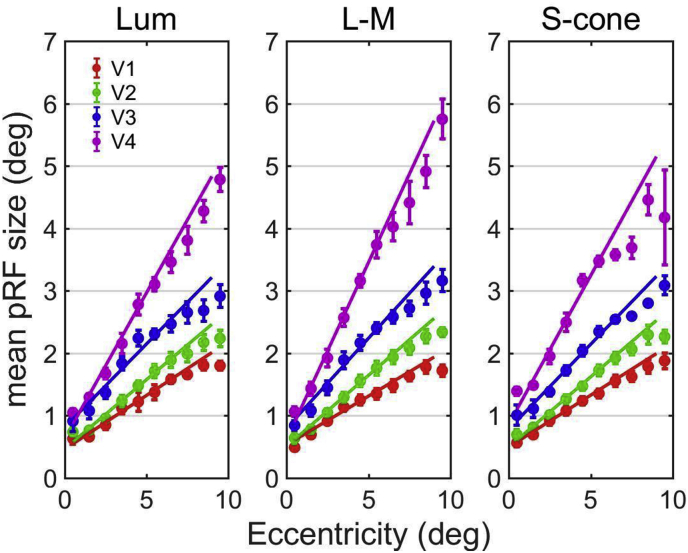
pRF sizes from the CSS pRF model. pRF sizes plotted as a function of eccentricity. Mean pRF sizes across subjects (n = 6), with standard error bars, are shown across eccentricities for each visual area (V1 (red), V2 (green), V3 (blue) and V4 (magenta)), with line of best fit shown for each visual area. Individual plots are provided for each condition (from left to right: luminance, L-M, and S-cone).

**Table B.1:** Mean proportion of variance explained for each condition (Luminance, L-M, and S-cone) in the original pRF model and in the CSS pRF model. Shown for each subject along with the group means and SEM.

Subject Number	Luminance		L-M		S-cone	
	Original pRF model	CSS pRF model	Original pRF model	CSS pRF model	Original pRF model	CSS pRF model
1	0.365	0.365	0.428	0.431	0.398	0.403
2	0.379	0.380	0.498	0.500	0.417	0.417
3	0.258	0.259	0.389	0.390	0.323	0.326
4	0.431	0.433	0.494	0.495	0.405	0.404
5	0.297	0.295	0.380	0.379	0.293	0.296
6	0.357	0.359	0.425	0.426	0.364	0.363
**Mean**	**0.348**	**0.349**	**0.435**	**0.437**	**0.367**	**0.368**
**SEM**	**0.025**	**0.025**	**0.021**	**0.021**	**0.020**	**0.020**

## Appendices

### Appendix B Compressive Spatial Summation (CSS) pRF model

The pRF data produced using the CSS pRF model (Kay et al., 2013) are plotted in Figure B.1, with pRF sizes plotted as a function of eccentricity for each chromatic condition, and separated by visual area (see legend). As with the pRF data presented in the Statistical analysis section ‘pRF mapping’, these data were analyzed using the foveal and peripheral eccentricity ROIs for each visual area, using a repeated-measures ANOVA with factors of visual area, eccentricity and condition. Mauchly's test of Sphericity was violated for the visual areas factor (χ^2^(5) = 14.515, *p* = 0.016), and therefore a Greenhouse-Geisser correction was applied prior to the interpretation of the visual area factor and associated interactions where Mauchly's test could not be run (i.e. three-way interaction and the interaction with condition).

Significant effects were observed for the factors of eccentricity (*F*(1,5) = 350.516, *p* = 10^−5^), and visual area (*F*(1.233,6.166) = 18.108, *p* = 0.004, Greenhouse-Geisser corrected), and a significant interaction was found between these two factors (*F*(3,15) = 4.718, *p* = 0.016). However, no significant effect of condition was observed (*F*(2,10) = 0.341, *p* = 0.719), and no significant interactions with the condition factor were found: condition and visual area (*F*(1.851,9.257) = 1.541, *p* = 0.263, Greenhouse-Geisser corrected), condition and eccentricity (*F*(2,10) = 3.302, *p* = 0.079), or for the three-way interaction between all factors (*F*(2.405,12.023) = 2.110, *p* = 0.159, Greenhouse-Geisser corrected).

In addition, we looked at whether the amount of variance explained by each of the models was different. Table B1 shows the mean variance explained for each subject in each condition (averaged across visual areas), for both the original pRF and the CSS pRF. Paired t-tests carried out between the condition pairs, and Bonferroni corrected to account for the multiple comparisons, found that there were no significant differences between any of the pairs.
